# Invariant object recognition based on extended fragments

**DOI:** 10.3389/fncom.2012.00056

**Published:** 2012-08-24

**Authors:** Evgeniy Bart, Jay Hegdé

**Affiliations:** ^1^Palo Alto Research Center, Intelligent Systems LaboratoryPalo Alto, CA, USA; ^2^Department of Ophthalmology, Vision Discovery Institute and Brain and Behavior Discovery Institute, Georgia Health Sciences UniversityAugusta, GA, USA

**Keywords:** form vision, illumination constancy, informative fragments, invariant recognition, mutual information

## Abstract

Visual appearance of natural objects is profoundly affected by viewing conditions such as viewpoint and illumination. Human subjects can nevertheless compensate well for variations in these viewing conditions. The strategies that the visual system uses to accomplish this are largely unclear. Previous computational studies have suggested that in principle, certain types of object fragments (rather than whole objects) can be used for invariant recognition. However, whether the human visual system is actually capable of using this strategy remains unknown. Here, we show that human observers can achieve illumination invariance by using object fragments that carry the relevant information. To determine this, we have used novel, but naturalistic, 3-D visual objects called “digital embryos.” Using novel instances of whole embryos, not fragments, we trained subjects to recognize individual embryos across illuminations. We then tested the illumination-invariant object recognition performance of subjects using fragments. We found that the performance was strongly correlated with the mutual information (MI) of the fragments, provided that MI value took variations in illumination into consideration. This correlation was not attributable to any systematic differences in task difficulty between different fragments. These results reveal two important principles of invariant object recognition. First, the subjects can achieve invariance at least in part by compensating for the changes in the appearance of small local features, rather than of whole objects. Second, the subjects do not always rely on generic or pre-existing invariance of features (i.e., features whose appearance remains largely unchanged by variations in illumination), and are capable of using learning to compensate for appearance changes when necessary. These psychophysical results closely fit the predictions of earlier computational studies of fragment-based invariant object recognition.

## Introduction

We rarely encounter a given object under the same viewing conditions twice: the viewpoint, illumination, retinal size, and background all tend to differ from one encounter to the next. Yet, we have little difficulty in recognizing an object for what it is while ignoring the irrelevant image variations. How the visual system accomplishes this invariant recognition of objects (also referred to as perceptual constancy) has remained largely unclear (for reviews, see Walsh and Kulikowski, 1998; Wallis and Bulthoff, 1999; Christou and Bulthoff, 2000; Rolls, 2008; Biederman and Cooper, 2009). This is both because the underlying computational problems are profoundly difficult, and because experimental and computational studies have so far largely focused on understanding object recognition without these variations.

Previous studies have shown that the visual system can use local, informative image fragments of a given object, rather than the whole object, in order to recognize the object under constant viewing conditions (Ullman et al., 2002; Harel et al., 2007; Ullman, 2007; Hegdé et al., 2008; Lerner et al., 2008; Kromrey et al., 2010). Such image fragments are referred to as “informative fragments.” Computational studies indicate that this fragment-based approach is also beneficial specifically for invariant object recognition (Bart et al., 2004; Ullman and Bart, 2004), including for pose and illumination invariance.

These studies have identified two broad functional sub-categories of informative fragments useful for invariant recognition. One sub-category of fragments, referred to as “Invariant fragments,” are those local features whose appearance is largely resistant to variations in viewing conditions. For instance, the appearance of the hairline changes relatively little under variations of illumination, which therefore makes it useful for illumination-invariant face recognition. On the other hand, the appearance of many features changes significantly with viewing conditions, which makes them unsuitable as invariant fragments.

“Extended fragments” are a second sub-category of fragments useful for invariant object recognition. In contrast to invariant fragments, extended fragments do not require feature appearance to be stable under changes in viewing conditions. Instead, an extended fragment records the appearance of the given feature under all viewing conditions of interest. In principle, this may involve simply memorizing the appearance of a given feature under each set of viewing conditions. An extended fragment can then be used for recognizing the feature regardless of the viewing conditions. For instance, even though the appearance of a nose changes under variations of illumination, it can still be useful for recognition if one learns how a nose looks under various illuminations. Since extended fragments do not depend on feature appearance being resistant to viewing conditions, any feature can be used as an extended fragment. Therefore, extended fragments may provide more information to the visual system and thus achieve better performance. However, extended fragments may be more difficult to learn than invariant fragments. This is because, in order to use a feature as an extended fragment, one must somehow learn its appearance under the various viewing conditions.

The extent to which the human visual system actually uses either extended or invariant fragments in object recognition is largely unclear. The mechanisms by which we learn either type of fragments, and conditions under which they can be learned, are also unknown. While previous studies have addressed the question of feature learning in general [e.g., (Kobatake and Tanaka, 1994; Schyns et al., 1998; Wallis and Bulthoff, 1999; Wallis et al., 2009)], it is unclear whether and to what extent the mechanisms suggested by these studies can generalize to learning extended or invariant fragments, given that the nature of object fragments is fundamentally different from the features addressed by these studies (for details, see Ullman, 2007; Hegdé et al., 2008).

The present study focused on testing a specific hypothesis, namely that the visual system is capable of using extended and/or invariant fragments to help achieve a particular type of perceptual constancy, namely illumination-invariant object recognition. In particular, we varied the direction of illumination while holding all other viewing parameters, including other illumination parameters such as brightness or color of illumination, constant. Note that the general framework of extended and invariant fragments is not limited to illumination; in particular, it has been used for pose-invariant recognition as well (Bart et al., 2004; Ullman and Bart, 2004).

We have previously shown, in the context of the aforementioned informative fragments, that both humans and monkeys automatically learn the fragments when they learn new object categories, and can use the learned fragments to recognize whole objects (Hegdé et al., 2008; Kromrey et al., 2010). We therefore use a similar experimental design in the present study to characterize how the human visual system learns and uses extended and/or invariant fragments for illumination-invariant object recognition. We find that human subjects can automatically learn extended fragments when they learn new objects, and can use the learned extended fragments to recognize whole objects.

## Materials and methods

### Participants

Five adult volunteer human subjects (three females) with normal or corrected-to-normal vision participated in this study. All protocols used in this study conformed to the relevant regulatory standards, and were approved in advance by the Human Assurance Committee of the Georgia Health Sciences University, where the psychophysical experiments were carried out. All subjects gave informed consent prior to participating in the study.

### Stimuli

**Figure 1:**
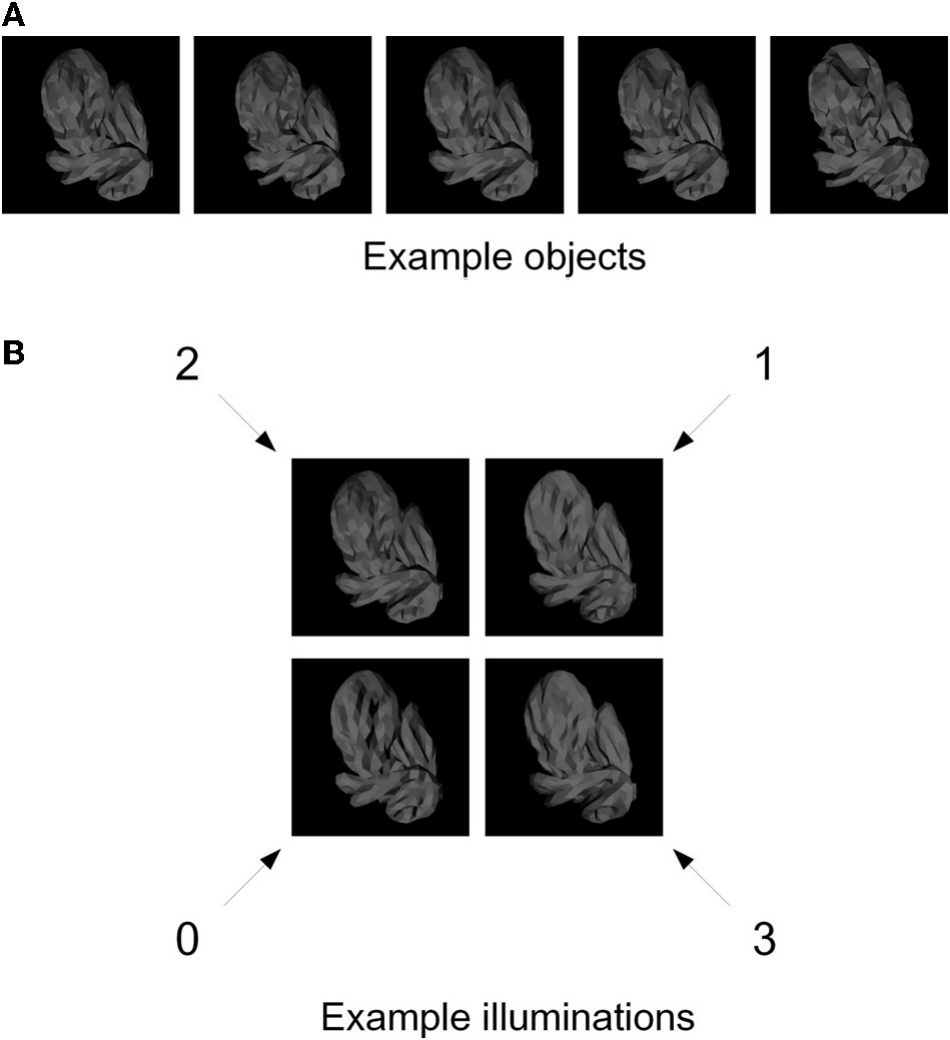
**Training stimuli. (A)** Five example digital embryos from our training set. All five embryos are shown under the same illumination. Note that the embryos are perceptually similar enough that distinguishing among them is not trivial. **(B)** The four directions of illumination used in our experiments. The directions are denoted by arbitrary numbers: 0 (illuminated from bottom left), 1 (from top right), 2 (from top left), and 3 (from bottom right). The same digital embryo is shown under the four illumination directions to illustrate the appearance changes induced by changes in illumination.

We generated 50 novel, naturalistic virtual 3-D objects called “digital embryos” using a custom implementation of the Virtual Phylogenesis (VP) algorithm (Brady and Kersten, 2003; Hegdé et al., 2008; Hauffen et al., in press). All 50 embryos were descendants of the same parent object, and thus constituted a single naturalistic “category.” The overall appearance of all objects was similar, with relatively small variations distinguishing individual objects from each other, so that distinguishing one embryo from another was nontrivial (see, e.g., Figure 1A). It is important to note that these shape variations were not imposed externally, but rather arose randomly during VP. To the extent that VP simulates the natural processes of morphogenesis and phylogenesis, these variations can be considered naturalistic (Hegdé et al., 2008; Hauffen et al., in press).

For each embryo, we generated four different images, corresponding to four different directions of illumination (illuminated from top left, top right, bottom left, and bottom right; see Figure 1B) using the 3DS Max graphics toolkit (Autodesk, Inc., San Rafael, CA).

### Fragment selection

Difference-of-Gaussians (DoG) interest points were located in each embryo image as described by (Lowe, 2004). A 20 × 20-pixel window around each interest point was extracted to form a candidate fragment.

For each fragment, the mutual information (MI) was computed. The MI *I(F; L)* between the fragment *F* and the object identity label *L* is defined as *I(F; L)* = *H*(*L*) − *H*(*L* | *F*), where *H*(*X*) is the entropy of the random variable *X* and measures the uncertainty in the value of *X*. Thus, *H*(*L*) is the uncertainty in the identity label of the given image in the absence of any information, and *H*(*L* | *F*) is the uncertainty in the identity given the information in the fragment *F*. Therefore, MI of the fragment *F* measures how much the uncertainty about object identity decreases by using the given fragment.

In practice, the MI can be computed by using the expression

(1) $$I(F;L)=\sum_{f,l}p(f,l)\log\frac{p(f,l)}{p(f)p(l)}.$$

The quantities of interest *p*(*f*, *l*), *p*(*f*), and *p*(*l*) can be evaluated from the training images, i.e., the set of images used as input to the fragment selection process. For example, the quantity *p*(*F* = 1) is the probability that a given fragment is present in an image. Similarly, the quantity *p*(*F* = 1, *l*) is the probability that the fragment is present in an image of object *l*.

**Figure 2:**
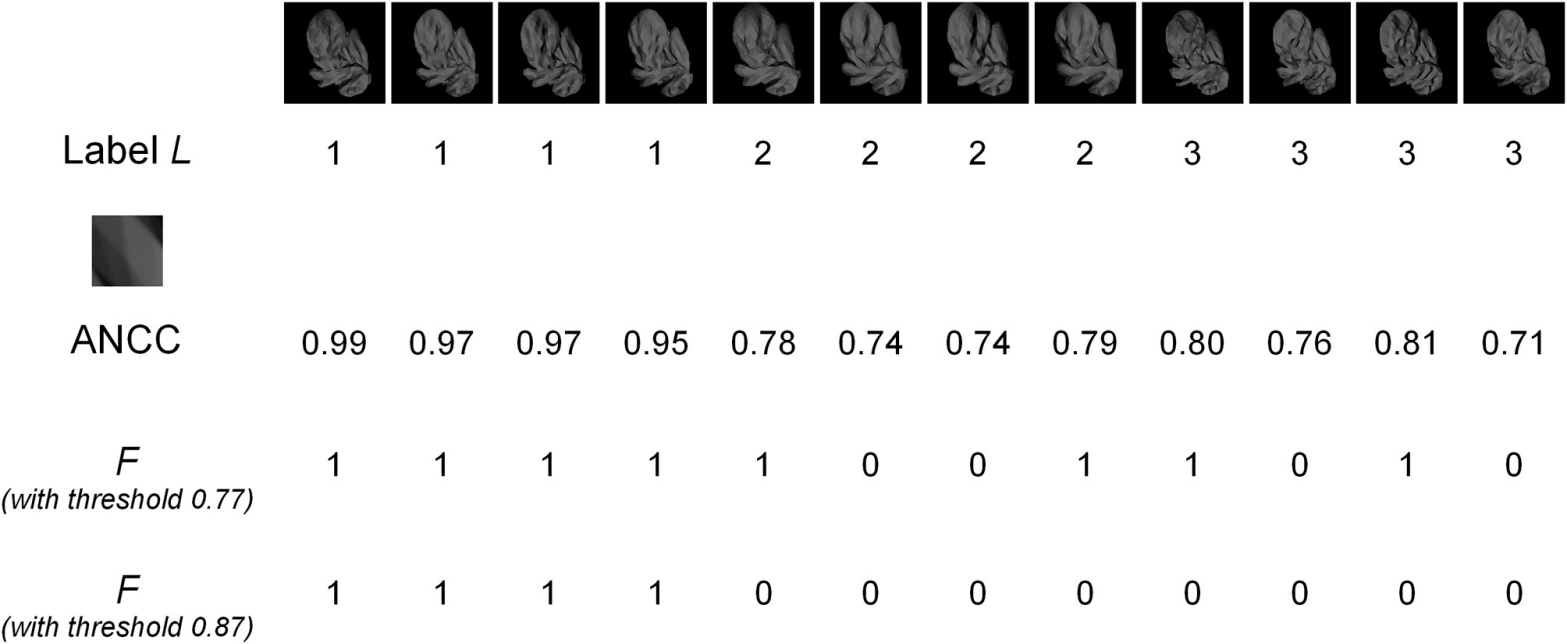
**An example of MI computation.** The top row shows images of three different objects (labeled 1, 2, and 3), with four different images for each object. The second row shows the object labels. The MI of the fragment shown (enlarged) in row three was computed. The fourth row shows the ANCC values for this fragment in each image. Using a threshold value of 0.77 gives the presence/absence values of the *F* variable shown in the fifth row. Since there are four 0's in this example, out of 12 total observations, we get an empirical estimate *p*(*F* = 0) = 4/12 = 1/3. Similarly, the following estimates can be obtained: *p*(*F* = 1) = 2/3; *p*(*L* = 1) = 1/3; *p*(*L* = 2) = 1/3; *p*(*L* = 3) = 1/3; *p*(*F* = 0, *L* = 1) = 0; *p*(*F* = 0, *L* = 2) = 1/6; *p*(*F* = 0, *L* = 3) = 1/6; *p*(*F* = 1, *L* = 1) = 1/3; *p*(*F* = 1, *L* = 2) = 1/6; *p*(*F* = 1, *L* = 3) = 1/6. Substituting these values into Equation (1), we get MI = 0.25. The ANCC values need to be computed only once, but the *F* values need to be recomputed for every threshold. For example, for the threshold setting of 0.87, the *F* values in row six are obtained, giving MI = 0.92. If the ANCC values are sorted in increasing order, the following sequence is obtained: 0.71, 0.74, 0.74, 0.76, 0.78, 0.79, 0.80, 0.81, 0.95, 0.97, 0.97, 0.99. Any threshold in-between two consecutive ANCC values will result in the same *F* values and therefore in the same MI. Therefore, in this example only 11 representative threshold values need to be evaluated to select the optimal threshold.

The presence of fragments in images was determined by using the absolute value of normalized cross-correlation (ANCC), as previously described in (Bart et al., 2004; Ullman and Bart, 2004). Briefly, to determine whether a given 20 × 20-pixel fragment *V* was present in a given image *X*, ANCC was first computed between the fragment and all 20 × 20-pixel windows in the image. The highest ANCC value was taken; this highest value is denoted *A*(*V*, *X*). If *A*(*V*, *X*) was above a pre-determined threshold, the fragment was considered present in the image (*F* = 1); otherwise, it was considered absent (*F* = 0). Thus, a 20 × 20-pixel fragment and a threshold determine the variable *F* and can be used to compute MI. The appropriate value of the threshold itself was determined by considering multiple threshold values for each fragment and selecting the threshold that maximized MI, as in (Bart et al., 2004; Ullman and Bart, 2004). ANCC values themselves were computed as follows. For two 20 × 20-pixel windows V, W, normalized cross-correlation is defined as

(2) $$\begin{array}{l} NCC(V,\;W)\\ =\frac{\frac{1}{20\times 20}\sum_{x=1}^{20}\sum_{y=1}^{20}(V[x,y)]-\bar{V})(W[x,y]-\bar{W}}{\sigma V\sigma W}, \end{array}$$

where *V*[*x*, *y*] is the pixel value at position (*x*, *y*) in the window *V*, $\bar{V}$ is the average of all pixel values in *V*, σ_*V*_ is the standard deviation of the pixel values, and similarly for *W*. Normalized cross-correlation has values between +1 and −1; the value +1 indicates perfect correlation, −1 indicates perfect anti-correlation, and 0 indicates no correlation. The ANCC therefore has values between 0 and 1, with lower values indicating weaker correlation and higher values indicating stronger positive or anti-correlation (in practice, anti-correlation rarely occurs in our images; data not shown). An example of MI computation and threshold selection is given in Figure 2.

There are two ways to use ANCC to determine a fragment's presence in a given image. One is to render the fragment under a fixed illumination (say, illumination 0) and use the rendering at this illumination to compute ANCC, regardless of which illumination the given image is in. Mathematically, we set *F* = 1 if the single template's ANCC value is above the threshold and compute MI using Equation (1). Of course, if the fragment appearance changes across illuminations, the results will be poor when the fragment illumination is different from the image illumination. This method of computation therefore implicitly assumes that the fragment's appearance is invariant to viewing conditions. When fragments are used in this manner, they are called “invariant fragments,” and MI computed in this manner is called “Invariant MI” and denoted by *I*_inv_. See Bart et al. (2004), Ullman and Bart (2004) for details.

**Figure 3:**
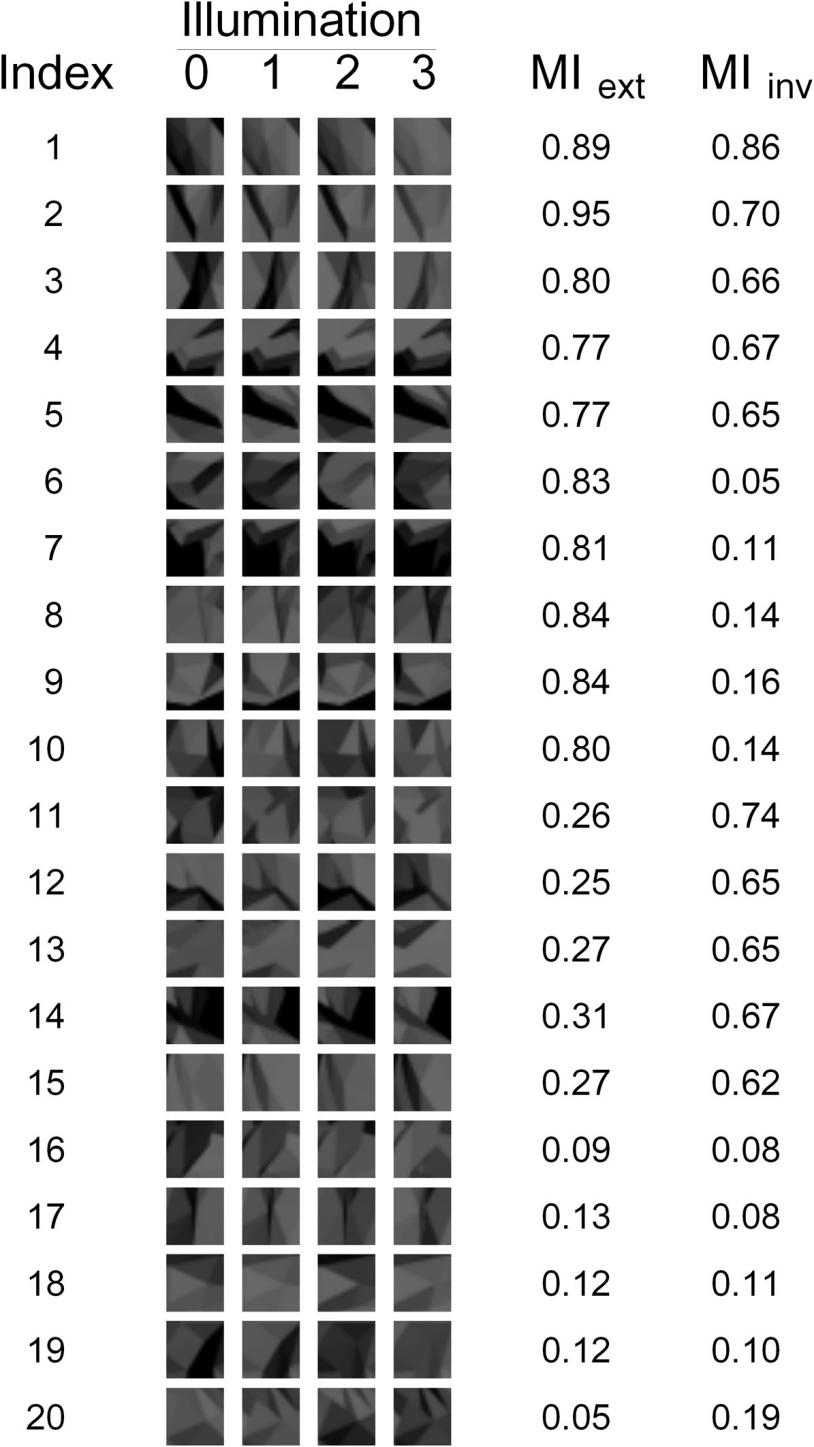
**The 20 fragments used in our experiments.** The appearance of each fragment under each of the four illuminations is shown, as well as the corresponding Extended and Invariant MI values (MI_ext_ and MI_inv_, respectively).

A second method of using ANCC to determine the presence of a fragment in a given image is to learn the appearance of each fragment under all illuminations in question. Computationally, this requires rendering and storing for each fragment the four templates, one for each illumination, as illustrated in Figure 3. In a biological system, this could be achieved by learning the appearance of a given feature in a given set of training examples. Given an image in a particular illumination, all four templates are matched to it using ANCC, and the best-matching template is selected in order to calculate the similarity. Mathematically, we set *F* = 1 if the maximal ANCC value over all four templates is above the threshold. The advantage of this method is that matching across illuminations is no longer necessary. In most cases, the template with the best ANCC value will automatically be the one that matches the image illumination (Bart et al., 2004; Ullman and Bart, 2004), thus eliminating comparison across illuminations. This generally results in much better similarity estimates and improved recognition performance (Bart et al., 2004; Ullman and Bart, 2004). The disadvantage is that training examples are needed, and the learning process may be difficult. When fragments are used in this manner, they are called “extended fragments,” and MI computed in this manner is called “Extended MI” and denoted by *I*_ext_. See Bart et al. (2004); Ullman and Bart (2004) for details.

For each candidate fragment, both *I*_ext_ and *I*_inv_ were calculated. Four “goodness” measures were defined as follows:

- G_1_ = *I*_ext_ + *I*_inv_ favors fragments that have high Extended MI and high Invariant MI.
- G_2_ = *I*_ext_ − *I*_inv_ favors fragments that have high Extended MI and low Invariant MI.
- G_3_ = −*I*_ext_ + *I*_inv_ favors fragments that have low Extended MI and high Invariant MI.
- G_4_ = −*I*_ext_ − *I*_inv_ favors fragments that have low Extended MI and low Invariant MI.

For each of these measures, the fragments were sorted according to the decreasing value of the measure. Note that *G*_3_ = −*G*_2_; the reason to use both is that we wanted to have fragments with high *I*_ext_ and low *I*_inv_, as well as fragments with low *I*_ext_ and high *I*_inv_. This allowed us to disassociate between *I*_ext_ and *I*_inv_ and determine how each separately affects the performance. Similarly, *G*_4_ = −*G*_1_; the reason to have *G*_1_ was to assess any additive effects of *I*_ext_ and *I*_inv_, while the reason to have *G*_4_ was to assess the performance of uninformative fragments. The top 20 fragments were selected for each measure, subject to the constraint that a fragment's visual similarity (as measured by ANCC) to any previously selected fragment could not exceed 0.9. This resulted in a total of 80 fragments.

The 20 fragments selected by measure G_1_ are shown in Figure 7. Note that there are still many fragments in this set that are visually similar to each other and thus redundant. Therefore, five non-redundant fragments were selected from this set manually by the authors (fragments 1–5 in Figure 3 and fragments 3, 4, 14, 17, and 20 in Figure 7). Similarly, five non-redundant fragments out of each subset of 20 were selected for the other goodness measures. This resulted in the final set of 20 non-redundant fragments shown in Figure 3. Note that this final set contains five fragments selected by each of the four goodness measures.

### Training in illumination-invariant object recognition

**Figure 4:**
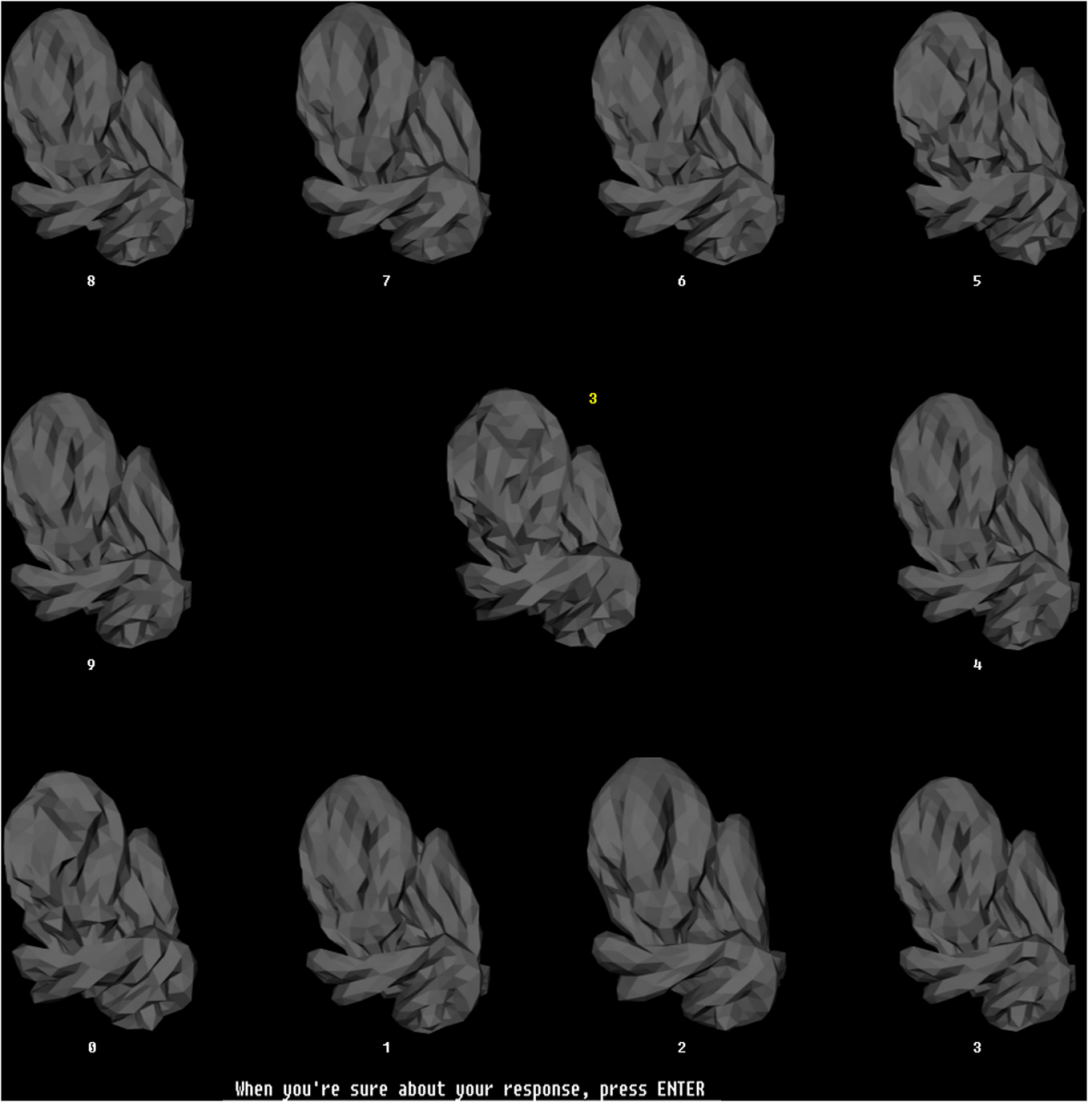
**The training paradigm.** This figure illustrates the configuration of stimuli during a typical trial during the training phase. During each trial, a randomly selected sample embryo was shown in the center in a randomly selected illumination. The 10 test stimuli were shown simultaneously arrayed along the periphery of the screen. The illumination was the same across all sample stimuli, but was different from the illumination of the sample embryo. The test embryos were assigned randomly to numbered locations (white numbers). One of the test embryos was the same object as the sample embryo, but at a different illumination. The subjects had to identify the test embryo that matched the sample embryo, and enter the number of this test embryo using the computer's keyboard, which then appeared as a yellow number next to the sample embryo. Note that this task required the subjects to generalize across the illuminations. The subjects pressed another key to finalize their response. After the subjects finalized their response, they received visual feedback (not shown), along with the correct response, if the subject's response was incorrect. Subjects had unlimited time both to perform the task and to examine the subsequent feedback. The stimulus configuration shown subtended 26° × 26° during the actual experiments.

**Figure 5:**
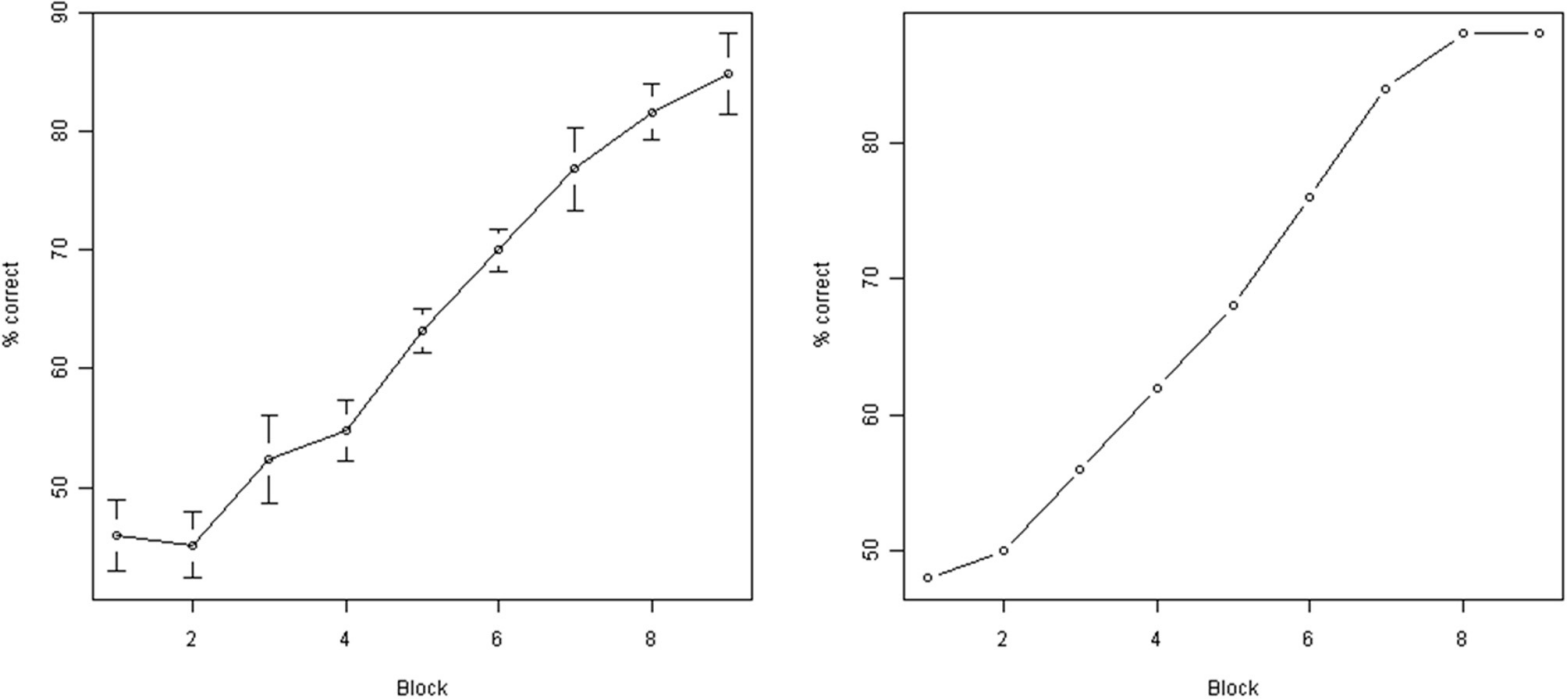
**Subject performance during training.** Y axis: % correct responses during a single block (50 individual trials). X axis: block number. Left: average across all subjects (error bars indicate standard error of the mean). Right: performance of a single representative subject (subject M00). As can be seen, the performance improved significantly as a result of training.

Except where noted otherwise, the procedures used in the psychophysical training phase (this section) and testing phase (see next section) of the experiment were identical to those described by us previously (Hegdé et al., 2008). Briefly, during the training phase, we trained the subjects to recognize individual digital embryos across illuminations using a simultaneous match-to-sample task. In this task, the subjects had to match a single sample embryo at one illumination at the center of the screen to an array of ten test embryos at another illumination arranged along the periphery of the screen (Figure 4). The subjects were allowed unlimited time to examine the images and arrive at a decision. Once the subjects reported their decision using a key press, visual feedback was provided (including the correct response, if the subject's response was wrong). The subjects had unlimited time to re-examine the display in light of the feedback. During initial training, the subjects were not required in any way to learn the fragments, nor were they even told of their existence. The performance was monitored across the training blocks (Figure 5). After a subject's performance remained asymptotic at above-chance levels for at least three sequential training blocks of 50 trials each (binomial tests, *p* < 0.05), the subjects moved to the testing phase (see below). All the subjects achieved asymptotic learning within 10 blocks (not shown). To minimize day-to-day forgetting of the learned objects, each subject carried out up to 50 “refresher” training trials at the start of each testing day. Note that during these refresher trials the subjects were aware of the existence of fragments, although they still weren't explicitly asked to learn them.

### Testing illumination-invariant object recognition using fragments

**Figure 6:**
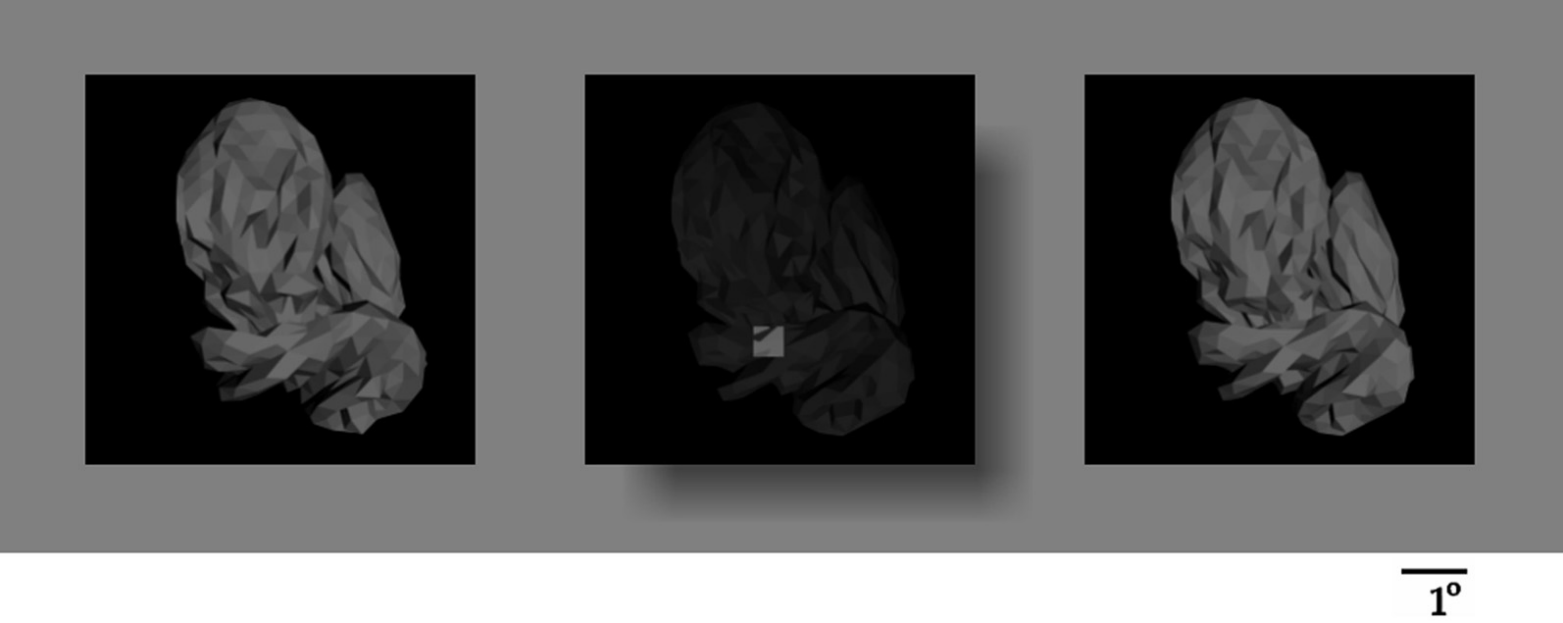
**The testing paradigm.** A composite object (center) and two test objects (left and right) were presented simultaneously during each trial. The composite object was occluded by a translucent surface with a hole, such that only the given object fragment was visible, unoccluded, through the hole, and the location of the fragment relative to the overall object was apparent through the translucent occluder. Subjects were informed that only the fragment, but not the darkened remainder of the composite object, was useful for the task. The fragment in the composite object was always in illumination 0, and both test embryos were always at illumination 3. The fragment was present in one of the test embryos, and absent from the other (“positive” and “negative” embryos, respectively). The location of the two test embryos was shuffled randomly from one trial to the next. Subjects had to report, using a key press, whether the positive test embryo was to the left or right of the composite object.

During the testing phase, the subjects performed an object identification task on the sole basis of a given fragment. In each trial, a composite object showing a sample fragment at illumination 0 was displayed at the center of the screen. Two test embryos at illumination 3 abutted the composite object. All stimuli were presented simultaneously for 3000 ms (Figure 6). Only one fragment in the composite object was clearly visible (see below). This fragment (called the “sample fragment”) was also present in one of the test embryos (“positive embryo,” presented on a randomly chosen side during a given trial) and absent from the other test embryo (“negative embryo”). Following a 200 ms random noise mask, subjects had unlimited time to indicate, based on the sample fragment in the composite object, whether the composite object was the same as the left test embryo or the right test embryo.

**Figure 7:**
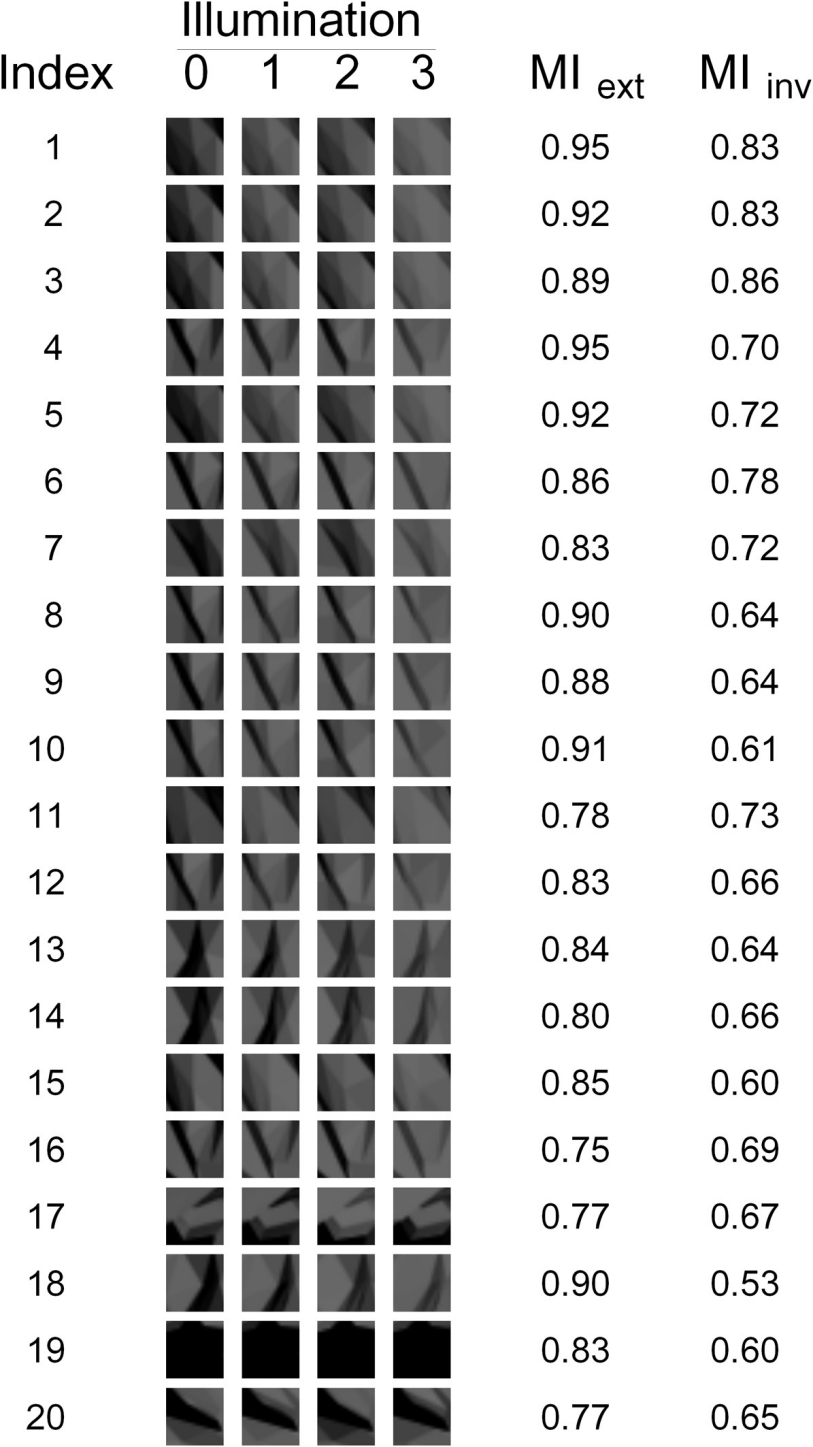
**The 20 fragments selected by the G_1_ measure.** The appearance of each fragment under each of the four illuminations is shown, as well as the fragment's Extended and Invariant MI.

The composite object was generated by graphically overlaying the sample fragment over a randomly drawn “background” embryo. The composite object was shown to the subject behind a rectangular translucent occluder with a hole, so that only the sample fragment (0.53° × 0.53°) was visible unhindered through the hole in its proper position on the object, whereas the rest of the object appeared as a faded “background” (see Figure 6). This design helped ensure that the subjects saw the sample fragment in its proper spatial context. This design is better than presenting the sample fragment by itself without the spatial context, because it minimizes the possibility that the subject may have to use semantic and spatial cues (e.g., configural cues, such “the corner of the left eye”) to help perform the task.

Subjects were informed that only the unoccluded fragment of the composite object was useful for the task, and that the faded background portion of the composite object (i.e., the portion visible behind the translucent occluder) was randomly selected, so that they would not be able to perform the task above chance levels using the background object.

Two different test objects (called “test embryos”) were shown on either side of the composite object. Whole objects, rather than just fragments, were used as test objects to help ensure that (1) the task involved object identification, as opposed to simple visual matching of individual fragments, and (2) task required only implicit perceptual learning and not declarative (or explicit) association between a fragment and an object.

A sample fragment and two test embryos (one positive and one negative) constitute a “testing configuration.” For each fragment in Figure 3, five embryos in which the fragment was most active, and five embryos in which it was least active, were selected. This activation level was measured by finding the highest ANCC value among all illuminations of a given embryo. All 25 possible testing configurations for each of the 20 fragments were created, resulting in 500 total testing configurations. This choice of testing configurations was motivated by the following considerations. The test embryos need to be visually distinguishable on the basis of the sample fragment; otherwise, the trial will be meaningless as the fragment will provide no information as to the correct answer. Embryos with highest fragment activation were compared to embryos with the lowest fragment activation to maximize this visual distinguishability. We used five embryos of each type, because fewer than 25 configurations per fragment might be insufficient to ensure thorough testing, while more than 25 configurations would make the testing too long and laborious for the subjects.

No feedback was provided during testing. Each fragment was presented over six randomly interleaved repetitions for each subject, so each subject performed 3000 trials during the testing phase.

### Data analysis

The results were analyzed using scripts custom-written in R (r-project.org) and Matlab (Mathworks, Natick, MA, USA). Additional details of the analyses are provided in the “Results” section, where underlying rationale will be clearer.

## Results

Our study was aimed at testing the hypothesis that the human visual system *can* use invariant and/or extended fragments to achieve invariant object recognition. During the testing phase of the experiment, the subjects had to determine which of the two test embryos contained the sample fragment (i.e., which one was the “positive” embryo). This task was difficult, because the sample fragment was presented in illumination 0, while both test embryos were presented in illumination 3. This difference in illumination induced a significant change in appearance (see, e.g., Figure 3) that the subjects had to compensate for in order to perform the task properly.

Several possible strategies for performing this task were evaluated. One possible strategy is that during each trial, the subject matches the fragment's visual appearance directly to both test embryos and selects the embryo that resembles the fragment more closely. Another possibility is that the subject discounts the illumination (for example, by somehow transforming the fragment into the embryo's illumination or vice versa) and then performs the visual comparison. A third possibility is that, as suggested by a computational model of invariance (Bart et al., 2004; Ullman and Bart, 2004) and our previous experiments (Hegdé et al., 2008; Kromrey et al., 2010), the subjects preferentially learn fragments that are useful for the object recognition task they were previously trained on. This usefulness can be measured objectively by using MI.

MI can be calculated under one of two hypotheses. One possibility is that the subjects assume illumination invariance, or preferentially seek out and exploit invariant fragments. The other possibility is that the subjects make no invariance assumptions and instead use learning to compensate for appearance changes across illumination by using extended fragments.

Five predictor variables corresponding to the strategies outlined above were computed for each testing configuration (i.e., the set of the sample fragment and two test embryos presented during a given trial):

1. M03 was the difference in visual similarity of the fragment to the “positive” and “negative” test images shown to the subject. Visual similarity was measured by ANCC, as described above. Denoting the fragment rendered in illumination 0 by *V*_0_, the positive test embryo rendered in illumination 3 by *X*^+^_3_, and the negative test embryo rendered in illumination 3 by *X*^−^_3_, M03 was defined as *A*(*V*_0_, *X*^+^_3_) − *A*(*V*_0_, *X*^−^_3_), where *A* was the ANCC value, as defined above. If the subjects used the naive strategy of direct matching by visual appearance, this M03 would be expected to correlate strongly with performance. Note that in practice, this strategy is likely to result in poor performance, since the fragment and the embryo images had different illuminations. This variable is also called Margin 0 → 3, which refers to the fact that a fragment in illumination 0 is matched to images in illumination 3.
2. M00 was the difference in visual similarity of the fragment to the “positive” and “negative” embryos rendered in illumination 0 (same illumination as the fragment). M00 was defined as *A*(*V*_0_, *X*^+^_0_) − *A*(*V*_0_, *X*^−^_0_), where *X*^+^_0_ was the positive test embryo rendered in illumination 0, and *X*^−^_0_ was the negative test embryo rendered in illumination 0. This variable is also called Margin 0 → 0. If the subjects mentally transformed the embryo images to illumination 0 and then used matching by visual appearance, this value would be expected to correlate strongly with performance.
3. M33 was the difference in visual similarity of the fragment, rendered in illumination 3 (same illumination as the embryo images) to the “positive” and “negative” images displayed. M33 was defined as *A*(*V*_3_, *X*^+^_3_) − *A*(*V*_3_, *X*^−^_3_), where *X*^+^_3_ was the positive test embryo rendered in illumination 3, *X*^−^_3_ was the negative test embryo rendered in illumination 3, and *V*_3_ was the fragment rendered in illumination 3. This variable is also called Margin 3 → 3. If the subjects mentally transformed the fragment image to illumination 3 and then used matching by visual appearance, this value would be expected to correlate strongly with performance.
4. Extended MI (MI_ext_) measured how useful the given fragment is for object recognition for subjects who use extended fragments.
5. Invariant MI (MI_inv_) measured how useful the given fragment is for object recognition for subjects who rely on the invariance of features across illumination.

Note that the first three variables, in general, change from one stimulus configuration to the next, while the last two variables have the same value for all 25 configurations involving a single fragment.

**Figure 8:**
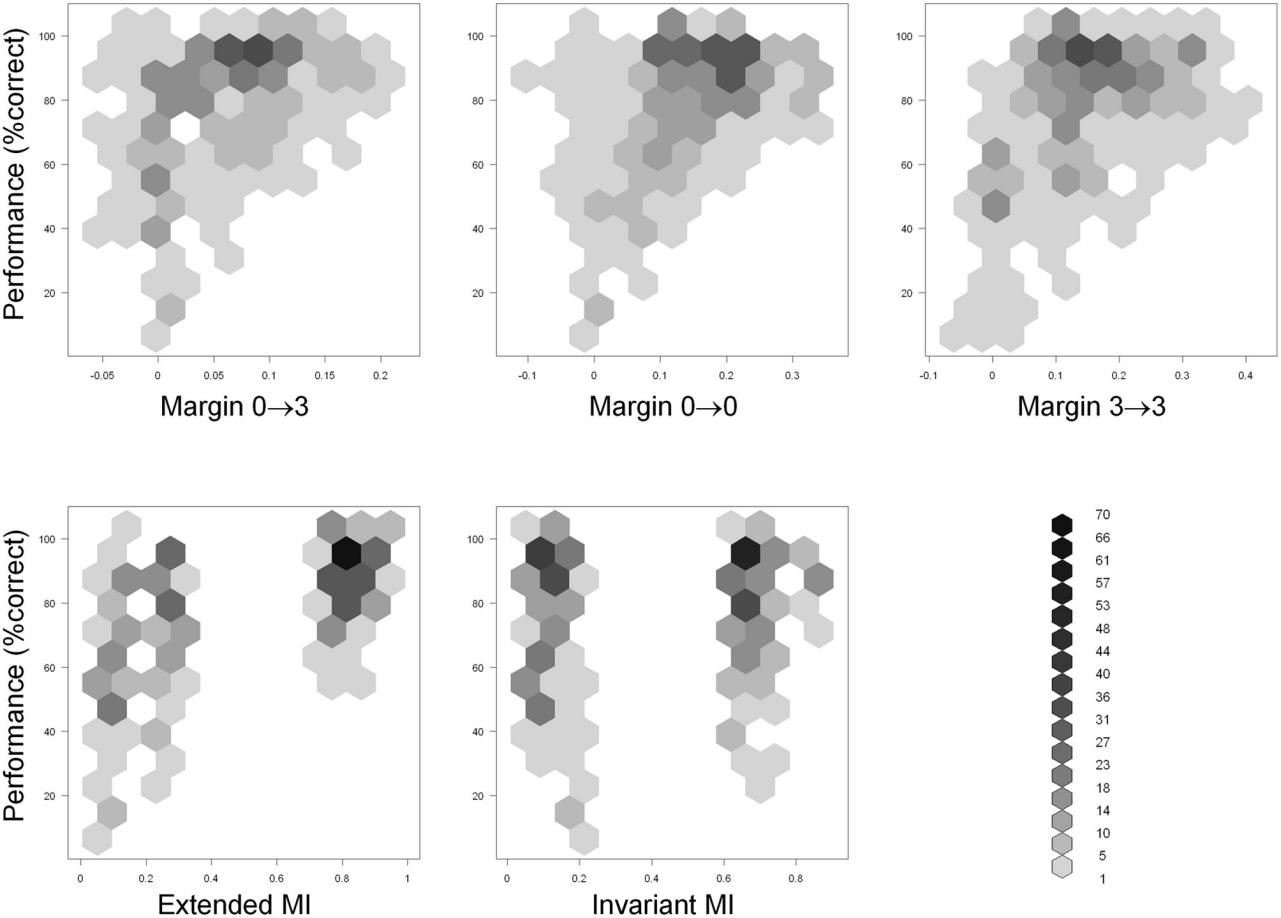
**Scatter plots of performance (Y axis) with the five predictor variables (X axis) defined in the “Results” section.** Hexagonal binning was used due to a large number of overlapping points. The depth of shading of each bin indicates the number of points that fall in it, according to the legend at the bottom right.

**Figure 9:**
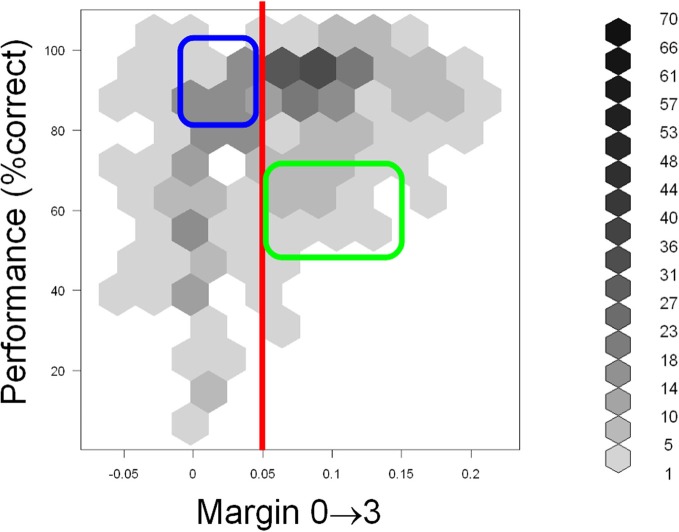
**Scatter plot of performance (Y axis) with the M03 variable (X axis) defined in the “Results” section.** Hexagonal binning was used due to a large number of overlapping points. The depth of shading of each bin indicates the number of points that fall in it, according to the legend on the right. The red line indicates the “visual recognizability” threshold, defined in the “Results” section. Note that testing configurations remained discernible even below this threshold (blue rectangle). However, subjects systematically underperformed in some highly recognizable configurations (green rectangle), indicating that factors other than visual recognizability affected performance. See text for details.

Scatter plots of performance with the five predictor variables are shown in Figure 8. Examination of performance averaged across all subjects revealed that the subjects systematically underperformed in many configurations despite abundant visual cues. This suggests (although does not, by itself, prove) that visual appearance alone was insufficient to explain the subjects' performance. To help discern whether this is indeed the case, we defined a configuration to be “visually recognizable” if the margin M03 was above 0.05 (note that the absolute values of normalized correlation range from 0 to 1). This threshold is shown as a red vertical line in Figure 9. The underlying intuition was that this amount of visual difference is easily detectable by human observers and can therefore be interpreted reliably. This intuition is confirmed by the fact that 67 configurations with M03 *less than* 0.05 were recognized correctly in over 80% of the trials (blue rectangle in Figure 9). In other words, even smaller margins were sufficient to allow reliable recognition. However, there were 49 configurations with a margin above 0.05 whose recognition rate was between 50 and 70% (green rectangle in Figure 9). Note that 50% recognition is expected by chance. In other words, even though these configurations contained sufficient visual cues to perform the task, the subjects systematically failed to do so. Similar results can be obtained using M00 or M33 to define visual recognizability instead of M03 (see Figure 8). These informal considerations support, although do not by themselves prove, the notion that factors other than visual recognizability significantly affect subjects' performance.

To rigorously analyze the intuition presented above, we fitted a linear regression to the data that accounted for the average performance in terms of the aforementioned five independent variables. An examination of the fitted model revealed that MI_ext_ was the only independent variable that contributed significantly to the fit (Table 1). This contribution was highly significant (*p* = 1.5 × 10^−14^, *F*-test). The contributions of the three visual variables (M03, M00, and M33), as well as the contribution of MI_inv_, were each statistically insignificant (*p* > 0.05).

**Table 1:** **Coefficients of linear regression for the five independent variables and the intercept term**.

**Variable**	**Estimate**	**Std. Error**	**Partial *r*^2^**	***p* value (*F*-test)**
Intercept	21.0	0.2	(Not Applicable)	2.0 × 10^−16^
M03	0.5	0.3	0.004	0.06
M00	0.5	0.4	0.003	0.15
M33	0.1	0.4	0.0001	0.71
MI_ext_	2.4	0.3	0.08	1.5 × 10^−14^
MI_inv_	0.4	0.2	0.004	0.06

With all terms included, the value of *r*^2^, the coefficient of determination, was 0.39.

We also compared the regression with the three purely visual variables (M03, M00, and M33) to regression with all five variables. Adding the MI-based variables had a highly significant effect (*p* = 7 × 10^−15^, *F*-test). In other words, even after accounting for the purely appearance-based factors given by the variables M03, M00, and M33, the MI-based variables explained a significant additional fraction of variance. In contrast, the performance of the two MI-based variables by themselves did not improve further after adding the three purely visual variables (*p* = 0.09, *F*-test). That is, the visual variables add no information beyond that already contained in the MI-based variables.

These analyses further support the conclusion that subjects do not rely on visual appearance alone, and can preferentially use extended fragments that are useful for the recognition task.

## Discussion

### Invariant object recognition based on fragments

Our results empirically confirm, for the first time, the hypothesis that the human visual system can use extended fragments to achieve invariant object recognition. The results found no support for the use of invariant fragments by the visual system. Note that this does not necessarily mean that the visual system cannot use invariant fragments for invariant object recognition under any circumstances; rather, it only shows that invariant fragments were not used in the current experiment. Nonetheless, it is worth noting that the statistical power of the sample was adequate enough to find affirmative evidence that the human visual system is capable of using extended fragments for invariant recognition. It is also worth noting that the fact that the visual system can use extended fragments under our experimental conditions does not necessarily mean that extended fragments are the universal, much less the sole, means by which the visual system achieves invariant object recognition in general, or illuminant-invariant recognition in particular (also see below).

The demonstration that the visual system is capable of using extended fragments is significant, for two main reasons. First, it provides the empirical “existence proof” for a hitherto theoretical idea. Second, as extensively noted by previous studies, fragment-based object approach is a substantially different approach to object recognition in general, and invariant object recognition in particular, than the conventional approaches based on whole objects [cf. (Wallis and Bulthoff, 1999; Christou and Bulthoff, 2000; Rolls, 2008; Biederman and Cooper, 2009; Wallis et al., 2009)]. Therefore, the empirical demonstration that the visual system can use fragments for this purpose opens important new avenues of future research for invariant object recognition in general, and illumination-invariant object recognition in particular (also see below).

### Illumination-invariant object recognition

Several key implications of our results for illumination-invariant object recognition are worth noting. First, if the subjects only used visual cues to perform the testing task, then the performance would be explained by the margin variables and would not be affected by MI. Since adding MI in fact improves the fit highly significantly (*p* = 7 × 10^−15^, *F*-test), we conclude that the subjects preferentially use informative fragments that are useful for the recognition task they were trained with. In contrast, uninformative fragments are neglected, even when sufficient visual information is available for accurate recognition.

Second, if the subjects compensated for illumination effects at the level of whole objects, then illumination of all features of a given object would be compensated for in a similar manner. The performance would thus depend only on how visually recognizable a given feature is after accounting for illumination. In practice, however, fragments with similar visual recognizability have dramatically different recognition rates (see “Results” for details). These considerations indicate that illumination compensation occurs on a feature level, rather than on a whole object level.

Finally, if the subjects assumed (implicitly or explicitly) that individual features were invariant to illumination, then the usefulness of individual features for recognition would be given by the Invariant MI. However, Invariant MI did not contribute significantly to explaining performance (*p* > 0.05, *F-test*). In contrast, the contribution of Extended MI, computed under the assumption that illumination is compensated for by extended fragments, rather than by assuming invariance, was highly significant (*p* = 1.5 × 10^−14^, *F*-test). Thus, subjects are highly unlikely to have assumed invariance, but rather must have compensated for viewing conditions by using extended fragments.

Note that for the training task we have used, computational simulations predict invariant features to perform much poorer than extended fragments. We cannot therefore conclude that subjects always use extended fragments. It is possible that when invariant features are sufficient to perform a task, those would be used instead of, or in addition to, extended fragments. However, as noted above, our results do provide an “existence proof” that subjects are capable of using extended fragments, and do use them when needed. Further work is necessary to determine under what conditions extended fragments can be learned and used. However, our “existence proof” is by itself an important conclusion, because using extended fragments is a nontrivial task.

Together, the above arguments support two main conclusions. First, illumination invariance is not achieved on a whole-object level. Rather, the illumination is compensated for feature-by-feature, with some features being preferred over others. The preferred features are those which support the recognition task, and their appearance variations are compensated for more carefully. Second, the subjects do not rely on invariance of individual features. Rather, they are capable of using extended fragments to compensate for appearance changes when necessary. Both conclusions fit closely with the computational model for invariant object recognition developed in Bart et al. (2004) and Ullman and Bart (2004).

### Leaning during training vs. prior learning

Using extended fragments to compensate for illumination requires familiarity with the visual appearance of a given object feature under various illuminations. This familiarity may be achieved by learning during the training process. Alternatively, this familiarity may be achieved by generalizing from previous visual experience, or may even be innate. The demonstration that subjects can use extended fragments at all is novel and interesting by itself, regardless of the exact learning mechanism used. We therefore did not attempt to establish the learning mechanism conclusively in this experiment.

In principle, some generalization from prior experience might have occurred in our experiment. For example, a corner may be recognizable as a corner under many different illuminations without dedicated training. However, it seems unlikely that such generalization would affect informative and uninformative fragments differentially, as in our experiment. There were no systematic visual differences between different fragments (see Figure 3). Moreover, the notion of MI itself is highly task-specific. For example, by computing MI for a different task where only two (rather than four) illuminations are used, the informativeness of fragments in Figure 3 changes dramatically. In particular, some of the uninformative fragments in Figure 3 become highly informative for this modified task (data not shown). The fact that subjects preferentially compensate for illumination changes of fragments informative for the given specific task, rather than for a number of possible alternative tasks, indicates that generalization from prior experience, if it exists, is modulated substantially by learning.

### Implications for the neural mechanisms for illumination-invariant object recognition

The neural mechanisms by which the visual system learns extended fragments, or uses them to achieve illumination-invariant object recognition, remain to be characterized. However, previous neuroimaging studies in human subjects have shown, using informative fragments, that the lateral occipital complex and the posterior fusiform gyrus are preferentially responsive to fragments with high MI values (Lerner et al., 2008), also see Harel et al. (2007). Both of these brain regions are known to play a central role in visual object recognition (Grill-Spector et al., 1999, 2000, 2001, 2004; Grill-Spector and Malach, 2004). Both of these regions have also been previously shown to play important roles in perceptual learning, albeit of whole visual objects (Gauthier and Tarr, 1997; Gauthier et al., 1998, 1999; Bukach et al., 2006; Wong et al., 2009). Taken together, these considerations suggest the possibility that these two brain regions play a key role in learning and/or using extended fragments for illumination-invariant object recognition.

It has been observed that object representations become both more selective and more invariant as they propagate upstream in the visual system (see, e.g., Rust and Dicarlo, 2010). This is thought to be a consequence of the hierarchical architecture of the visual system, where cells at higher levels pool input from several lower-level cells and thus become more tolerant of changes than each individual lower-level cell (Riesenhuber and Poggio, 1999). Our results are consistent with this view, because the features we have used are quite high-level, and are expected to be processed in high-level visual areas, and can therefore be expected to be quite tolerant of viewing conditions.

### Future directions

It is worth noting that our results, although highly statistically significant, only account for about 40% of the variability in the subjects' performance (Table 1). In the future, it would be interesting to determine what factors account for the remaining variability. One potential source of this residual variability is that our sample sizes, even with 3000 trials per subject (see “Materials and Methods”), were nonetheless relatively small from the statistical viewpoint. Using more testing configurations per fragment and repeating the experiments with more subjects and more trials per subject would help reduce the intrinsic randomness in the performance. Another potential source of variability is a scenario where the subjects learn features at a smaller scale than those extracted computationally, but learn different subsets of these smaller features. Although extracting such small features is easy computationally, it may present practical problems for our current experimental setup. This is because even the current features are small enough to cause visibility concerns. However, designing an experiment where smaller features are not useful for recognition, or using a different testing paradigm, may alleviate this problem.

Although the experiments in the current work addressed illumination invariance, it should be noted that our experimental setup can readily be used to test other types of invariant recognition, such as viewpoint (or pose) invariance, size (or scale) invariance, etc. This could be a particularly interesting direction for future work, especially since the underlying computations are fundamentally the same (Bart et al., 2004; Ullman and Bart, 2004) This is not necessarily to say, however, that the underlying neural mechanisms are the same. Indeed, given that the relevant visual features tend to be processed differently by the visual system (Felleman and Van Essen, 1991; DeYoe et al., 1994; Vuilleumier et al., 2002; Grill-Spector and Malach, 2004), the underlying neural mechanisms are likely to be substantially different. However, the fragment-based approach provides a common, rigorous conceptual framework for the experimental study of many different types of perceptual invariance.

### Conflict of interest statement

The authors declare that the research was conducted in the absence of any commercial or financial relationships that could be construed as a potential conflict of interest.
